# Prevalence and predictors of percutaneous injuries among health workers in Ghana: a cross-sectional study

**DOI:** 10.3389/fpubh.2025.1561098

**Published:** 2025-04-24

**Authors:** Philip Apraku Tawiah, Emmanuel Appiah-Brempong, Paul Okyere, Geoffrey Adu-Fosu, Mary Eyram Ashinyo, Florence Shine Edziah, Emmanuel Daitey Amesimeku, Priscilla Appiah Baffoe

**Affiliations:** ^1^Department of Occupational and Environmental Health and Safety, School of Public Health, College of Health Sciences, Kwame Nkrumah University of Science and Technology, Kumasi, Ghana; ^2^Department of Pharmacognosy and Herbal Medicine, School of Pharmacy, University of Health and Allied Sciences, Ho, Ghana; ^3^Department of Health Promotion and Disability Studies, School of Public Health, College of Health Sciences, Kwame Nkrumah University of Science and Technology, Kumasi, Ghana; ^4^Physiotherapy Unit, Diagnostic and Rehabilitation Directorate, Ho Teaching Hospital, Ho, Ghana; ^5^Department of Quality Assurance–Institutional Care Division, Ghana Health Service Headquarters, Accra, Ghana; ^6^Department of Maternal and Child Health, Gilling’s School of Global Public Health, University of North Carolina, Chapel Hill, NC, United States; ^7^Department of Medical Laboratory Sciences, School of Allied Health Sciences, University of Health and Allied Sciences, Ho, Ghana; ^8^Department of Nutrition and Dietetics, School of Biomedical and Allied Health Sciences, University of Ghana, Accra, Ghana; ^9^Department of Pharmaceutical Microbiology, School of Pharmacy, University of Health and Allied Sciences, Ho, Ghana; ^10^Department of Medical Microbiology, School of Medicine, College of Health Sciences, University of Ghana, Accra, Ghana

**Keywords:** needlestick injury, shift work, work pressure, hepatitis B and C virus infection, HIV infection, health personnel (MeSH), Ghana Africa

## Abstract

**Background:**

Percutaneous injuries (PI) persist as a prevalent healthcare issue, affecting over a third of healthcare workers worldwide on an annual basis. Globally, a few studies have documented the relationship between PI and factors like work pressure and shift systems. Additionally, limited evidence exists on how these factors contribute to this issue specifically in Ghana.

**Objective:**

The study examined exposure to PI and its predictors among health workers in Ghana.

**Methods:**

An analytic cross-sectional study involving multiple health facilities in the Greater Accra region was conducted between January 30 and May 31, 2023. A survey was carried out among 602 healthcare workers across 10 public and private hospitals. Study participants were selected using simple random sampling. Analysis was performed using Stata 15 software, and factors associated with PI were identified using log-binomial regression analysis, with a significance level set at *p* < 0.05.

**Results:**

The prevalence of PI was 26.9% (95% CI: 23.4–30.6%). More work experience [APR = 0.97 (0.94, 0.99)], being on a mix of day, evening and night shifts [APR = 1.69 (1.26, 2.27)], frequent experience of work pressure [APR = 1.32 (1.00, 1.75)], frequent [APR = 0.59 (0.40, 0.88)], and constant [APR = 0.55 (0.40, 0.7)] adherence to standard precautions were factors significantly associated with PI.

**Conclusion:**

Shift schedules and work pressure contributed to the substantial rate of PI among healthcare workers in Ghana. It is imperative for health authorities to establish and enforce safety policies prioritizing pressure reduction and fostering a safety-oriented culture across all shifts.

## 1 Background

In healthcare settings, percutaneous injuries (PI) continue to be a common occurrence, affecting over a third of healthcare workers worldwide on yearly basis (1). The Centre for Disease Control and Prevention (CDC) reported over 1 million incidents annually, accounting for 8% of injuries within hospitals in the United States. However, only half of these incidents have been formally documented (2, 3). Regrettably, at least 20 distinct highly contagious pathogens, such as hepatitis B virus (HBV), hepatitis C virus (HCV), and human immunodeficiency virus (HIV) are being transmitted through PI (4). Among healthcare professionals worldwide, the percentages of work-related infections associated with HBV, HCV, and HIV are 37, 39, and 4.4%, respectively (4). Furthermore, significant psychological complications such as depression and post-traumatic stress disorder (PTSD) may have enduring consequences on health workers after their exposure to these injuries (5).

The occurrence of PI is significantly affected by the environmental conditions in healthcare facilities. Health workers in operating rooms, emergency departments, intensive care units and on shift work schedules are more likely to experience PI (6). Additionally, studies suggest that increased work pressure, staffing shortages, a variety of invasive procedures, critical patient conditions, and multiple invasive interventions contribute to an elevated risk of injuries among nurses and other health workers in these environments (7). The strain of heavy workloads, fatigue, and decreased attentiveness resulting from work pressure can contribute to PI (8). Regarding shift work, a study observed a notable shift in PI rates among nurses on night shifts, initially decreasing but later increasing (9). These reasons explain why work pressure and shift work systems might be significant in the exposure to PI among health workers in Ghana and other developing countries. Also, health workers in developing nations face more challenging issues such as collapsed healthcare systems, inadequate facilities including insufficient personal protective equipment and waste disposal infrastructure, and the absence of infection control protocols, which increase the risk of exposure to PI (10, 11).

There is a plethora of research on PI among health workers both globally and in Africa; however, few studies have been conducted on the topic among health workers in Ghana, who still struggle with frequent accidental exposure to needlestick-and sharp-related injuries during their line of work (12, 13). Recent research in Ghana indicates that almost half of both health workers (47.0%) (14) and healthcare support staff (45.6%) (15) have experienced percutaneous injuries. Furthermore, the factors contributing to occupational NSI among healthcare workers (HCWs) in Ghana have not been adequately explored. For instance, there are limited data addressing the relationship between shift work, pressure at work, and the prevalence of PI among health professionals in previous studies conducted in Ghana and other African countries. In terms of policy, though there is a general occupational health and safety policy and guidelines for the health sector, there is none that specifically addresses percutaneous injuries. Therefore, this study investigated exposure to PI and its associated predictors including shift work and job pressure among health workers in the Greater Accra region of Ghana, with the aim of guiding future policy on exposure to percutaneous injuries among health workers in Ghana.

## 2 Methods

### 2.1 Study design, population and area

This study adopted a facility-based analytic cross-sectional design and quantitative methodology. The study population comprised doctors, nurses, midwives, medical laboratory scientists, and housekeeping staff employed in six public and four private hospitals located in the Greater Accra region of Ghana. These hospitals are major healthcare facilities in their respective districts, offering a range of services, including outpatient care, antenatal and family planning, dental care, eye care, laboratory services, ear-nose-and-throat care, radiology, dermatology services, and surgical procedures. The hospitals’ bed capacities varied from 50 to 500, and their workforce ranged from 77 to 579, comprising healthcare professionals and housekeeping staff. The Greater Accra region stands out for its high concentration of healthcare professionals, making up around 30.6% of Ghana’s total healthcare workforce, as reported in 2015 (16). As of 2021, the region had become the most populous in Ghana, with an estimated population of 5,455,692, representing about 17.7% of the country’s total population (17).

### 2.2 Sample size calculation

The Cochran formula (18), $N_{o}=\frac{z^{2}pq}{d^{2}}$ , determined the sample size for the study. Using z = constant for a 95% confidence interval given as 1.96, p = proportion of the population (46.0%) that was exposed to the outcome (PI) in a recent study conducted among health workers in Ethiopia (19), q = (1-p) and d = margin of error estimated as 5%, sample size, $N_{o}$ was estimated to be 382. After employing a design effect of 1.5, as recommended by previous studies (20), and an anticipated non-response rate of 10% to the sample size, we arrived at a final sample size of 630. A total of 602 health workers participated in the study, resulting in a response rate of 95.6%. The primary factor contributing to the failure to achieve a 100% response rate was the absence of financial compensation.

### 2.3 Sampling process

The research design incorporated a multi-stage sampling procedure. The Greater Accra region was purposively selected. Following this, a random selection of districts, hospitals, and study participants was done based on a proportional-to-size sampling approach, which ensured that the sample was representative of the population size. The Greater Accra region comprised 29 districts, including two metropolitan areas, 23 municipalities, and four districts. A total of 10 districts, making up over 30% of the total, were chosen for this research. The study’s sampling frame comprised 17 major hospitals, from which a random selection of 10 hospitals was selected for this study. Each district was represented by one major hospital, except in cases where districts had two or three major hospitals, where one was randomly selected. The selection of major hospitals was influenced by the 2021 annual outpatient department (OPD) attendance data from the District Health Information Management System (DHIMS). Participants were selected through a stratified random sampling approach, where their professions formed the strata, and random sampling was used to select individuals from each professional group.

#### 2.3.1 Inclusion and exclusion criteria

The study participants included doctors, nurses, midwives, medical laboratory personnel, and housekeeping staff. Additionally, individuals within these healthcare professions who had been employed at a hospital for at least 1 year were included based on the inclusion criteria. Conversely, health professionals, such as administrators, radiologists, dieticians, and health students, who were not specified in the inclusion criteria, were excluded from the study.

### 2.4 Study instrument and data collection

The data collection instrument used in this study was a structured questionnaire. While the entire questionnaire was purposefully crafted for this research, specific sections were adapted from a previously validated National Institute for Occupational Safety and Health, US Centre for Disease Control and Prevention’s Healthcare Workers Safety and Health Survey questionnaire (21). The questionnaire comprised a combination of closed-ended and open-ended questions and was structured into three sections: Section I focused on respondents’ socio-demographic and lifestyle characteristics; Section II addressed occupational-related factors; and Section III examined PI, encompassing seven, nine, and one question(s), respectively.

The questionnaire was pretested among 60 healthcare workers at Ho Teaching Hospital. Following the pilot phase, the questions were revised based on feedback from the study participants, faculty members in occupational health and safety, and key stakeholders from the Ghana Health Service. Data collection involved the distribution of paper-based questionnaires. Participants were given a self-administered questionnaire after a brief introduction and asked to complete it promptly. When participants encountered difficulties with questionnaire completion, research assistants conducted interviews to facilitate the process. Responses from the completed paper questionnaires were entered into a previously created electronic platform (Open Data Kit). The data were collected between January 30 and May 31, 2023.

### 2.5 Data management and analysis

The data were exported from the Open Data Kit electronic platform and imported into Stata SE version 15 (64-bit) statistical analysis software for cleaning and analysis. Before the analysis, a preliminary analysis of the data was conducted to detect and rectify any errors. Additionally, skewness and kurtosis tests were performed on the quantitative variables to determine their suitability for parametric or non-parametric tests. Descriptive statistics, such as frequencies and percentages, were used, while continuous variables were summarized using medians and interquartile ranges. The descriptive statistics for the independent variables, including socio-demographic and lifestyle characteristics, as well as occupational factors, are presented in a tabular format. In contrast, descriptive statistics for the dependent variable, PI, were illustrated using a bar chart. PI was evaluated using a single question on the frequency of exposure experienced by healthcare workers in the past year. The response options were categorized as “No” for “never” and “Yes” for exposure occurring once, twice, thrice, 4 times, or more than 5 times.

Initial associations between the prevalence of PI and independent variables were investigated using the chi-square test, Fisher’s exact test, and Mann–Whitney *U* test. The chi-square and Fisher’s exact tests were used to compare categorical variables. In contrast, the Mann–Whitney *U* test was used to compare a continuous variable across two distinct categorical groups. Additionally, the relationship between the independent variables and the prevalence of PI was validated using both bivariate and multiple log-binomial regression analyses. In the multiple log-binomial regression model, variables showing significance at or below a *p*-value of approximately 0.1 on the chi-square, Fisher’s exact, or Mann–Whitney *U* tests were included. The Variance Inflation Factor (VIF) was used to assess potential multicollinearity among the key explanatory variables. The final results indicated no evidence of multicollinearity (Mean VIF = 2.14, Minimum VIF = 1.33, and Maximum VIF = 3.28) after removing variables that exceeded the VIF threshold of 5.

## 3 Results

### 3.1 Socio-demographic and lifestyle characteristics of health workers

Table 1 presents a summary of the hospital workers sampled from 10 major hospitals in the Greater Accra region of Ghana. Out of the 602 health workers that participated in the study, more than half (55.2%) belonged to the nursing profession, and a little over one-tenth (10.8%) were orderly. The majority of the participants (51.5%) were within the 30–39-year-old range; and the median age was 32 years, with an interquartile range of 28–37 years. The dominant group of the participants (82.4%) were females. Also, a greater number of participants (46.8%) had worked for <5 years. The median work experience was 5 years (interquartile range, 3–12 years). Most study respondents, 553 (91.9%) had attained tertiary education. In addition, 87.5% of respondents worked with state-owned health facilities. Additionally, 81.1% of respondents worked for 5 days or less within a week.

**Table 1:** Socio-demographic and lifestyle characteristics influencing percutaneous injuries.

Characteristics	*N* (%)	Percutaneous injuries		*χ^2^*/t	*p*
		No	Yes		
Gender				0.36	0.550
Female	496 (82.4)	365 (73.59)	131 (26.41)		
Male	106 (17.6)	75 (70.75)	31 (29.25)		
Age				8.50	0.038*^a^
Younger than 30	210 (34.8)	142 (67.62)	68 (32.38)		
30–39	310 (51.5)	229 (73.87)	81 (26.13)		
40–49	66 (11.0)	55 (83.33)	11 (16.67)		
50 and older	16 (2.7)	14 (87.50)	2 (12.50)		
Highest educational level				0.00	0.950
Primary/Secondary	49 (8.1)	36 (73.47)	13 (26.53)		
Tertiary	553 (91.9)	404 (73.06)	149 (26.94)		
Occupation				4.30	0.366
Doctor	41 (6.8)	31 (75.61)	10 (24.29)		
Nurse	332 (55.2)	242 (72.89)	90 (27.11)		
Midwife	130 (21.6)	89 (68.46)	41 (31.54)		
Laboratory staff	34 (5.6)	29 (85.29)	5 (14.71)		
Orderlies	65 (10.8)	49 (75.38)	16 (24.62)		
Type of health facility				0.37	0.544
Private	75 (12.5)	57 (76.00)	18 (24.00)		
Public	527 (87.5)	383 (72.68)	144 (27.32)		
Years of experience				23.49	<0.001*
Less than 5	282 (46.8)	180 (63.83)	102 (36.17)		
5–10	108 (18.0)	90 (83.33)	18 (16.67)		
10 and above	212 (35.2)	170 (80.19)	42 (19.81)		
Working days in a week				2.20	0.138
5 and below	488 (81.1)	363 (74.39)	125 (25.61)		
Above 5	114 (18.9)	77 (67.54)	37 (32.46)		

*Significant variable (*p* < 0.05); ^a^*p*-values calculated from Fishers’ exact test. IQR, Interquartile range.

### 3.2 Occupational-related factors

The majority (51.2%) of the study participants worked overtime. More than half of the respondents (58.0%) occasionally experienced pressure from their work. Many of the participants (58.3%) experienced moderate amounts of stress, and almost two-thirds of them (65.8%) were understaffed in their department. In addition, the majority of participants (51.3%) were on a mix of day, evening, and night shifts. Moreover, a little over one-third of the study participants (37.5%) used prescribed protocols for work, and 29.7% used Personal Protective Equipment (PPE). Furthermore, the majority of participants (82.9%) had been trained on standard precautions, and almost two-thirds (65.8) adhered to standard precautions (Table 2).

**Table 2:** Occupational-related factors influencing percutaneous injuries.

Characteristics	Total (%)	Percutaneous injuries		*χ^2^*	*p*
		No	Yes		
Overtime				2.23	0.136
No	294 (48.8)	223 (75.85)	71 (24.15)		
Yes	308 (51.2)	217 (70.45)	91 (29.55)		
Shift				17.64	< 0.001*
Day only	293 (48.7)	237 (80.89)	56 (19.11)		
A mix of day, evening and nights	309 (51.3)	203 (65.70)	106 (34.30)		
Pressure				5.78	0.016*
Occasionally	349 (58.0)	268 (76.79)	81 (23.21)		
Frequently	253 (42.0)	172 (67.98)	81 (32.02)		
Stress				3.83	0.135
Almost no stress	12 (2.0)	8 (66.67)	4 (33.33)		
Moderate stress	351 (58.3)	267 (76.07)	84 (23.93)		
A lot of stress	239 (39.7)	165 (69.04)	74 (30.96)		
Understaffed				0.44	0.506
No	206 (34.2)	154 (74.76)	52 (25.24)		
Yes	396 (65.8)	286 (72.22)	110 (27.78)		
Use of prescribed protocols				19.52	< 0.001*
Rarely	38 (6.3)	27 (71.05)	11 (28.95)		
Sometimes	135 (22.4)	82 (60.74)	53 (39.26)		
Most of the time	226 (37.5)	185 (81.86)	41 (18.14)		
Always	203 (34.0)	146 (71.92)	57 (28.08)		
Use of PPE				8.14	0.043*
Rarely	142 (23.6)	102 (71.83)	40 (28.17)		
Sometimes	124 (20.6)	81 (65.32)	43 (34.68)		
Most of the time	179 (29.7)	143 (79.89)	36 (20.11)		
Always	157 (26.1)	114 (72.61)	43 (27.39)		
Adherence to standard precautions				13.73	0.001*
Sometimes	65 (10.8)	35 (53.85)	30 (46.15)		
Most of the time	141 (23.4)	106 (75.18)	35 (24.82)		
Always	396 (65.8)	299 (75.51)	97 (24.49)		
Training in standard precautions				1.66	0.197
No	103 (17.1)	70 (67.96)	33 (32.04)		
Yes	499 (82.9)	370 (74.15)	129 (25.85)		

*Significant variable (*p* < 0.05).

### 3.3 Prevalence of percutaneous injuries among health workers

**Figure 1:**
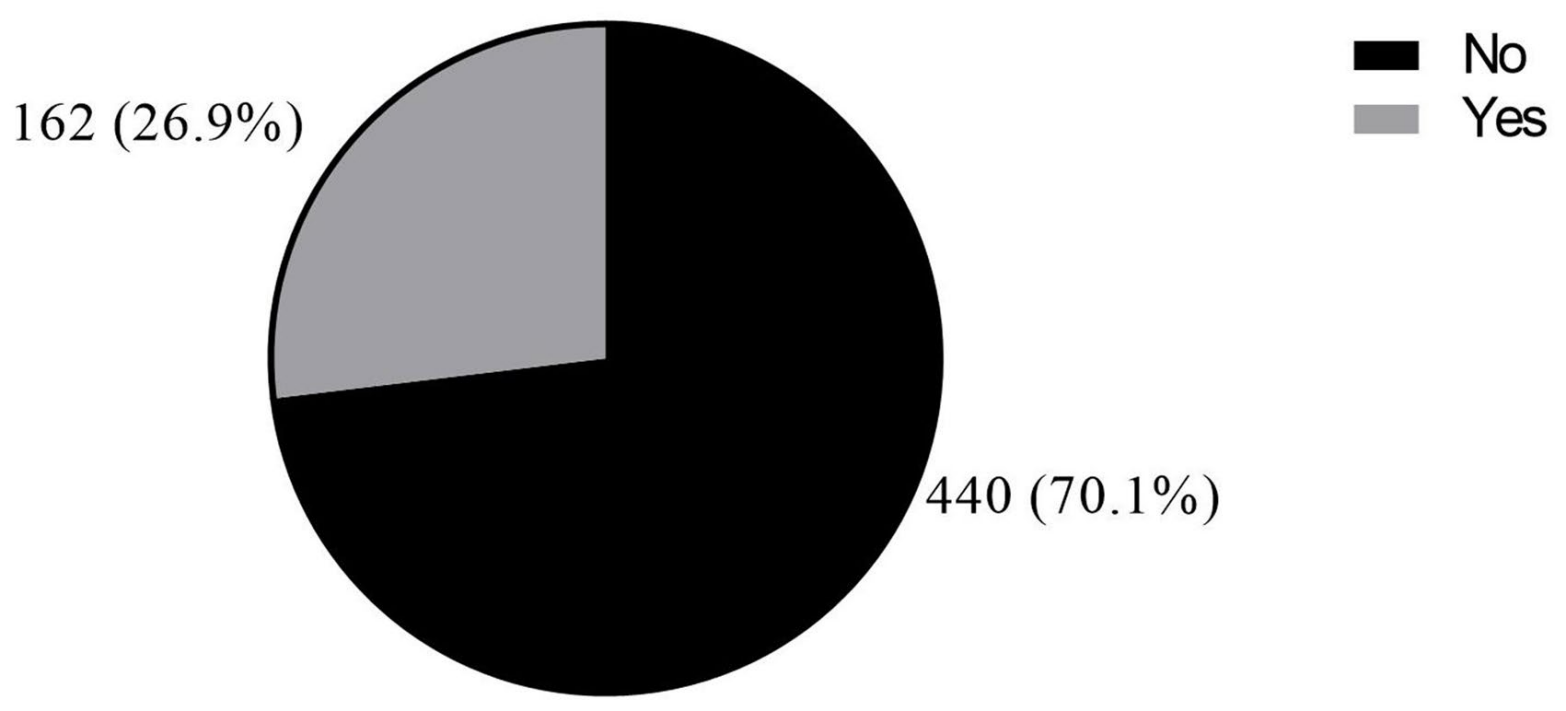
Percutaneous injuries among health workers.

Overall, a greater proportion of health workers, 162 (26.9%) (95% CI: 23.4–30.6%), were exposed to PI in the past year (Figure 1). The prevalence of PI was dominant among study participants who were younger than 30 years (32.4%). Additionally, it was also dominant among those who had <5 years working experience (36.2%) (Table 1).

### 3.4 Socio-demographic and lifestyle characteristics, and occupational-related factors influencing percutaneous injuries

A significant association was found between age (*t* = 3.04, *p* = 0.002), years of experience (*χ^2^* = 4.11, *p* < 0.001), and exposure to PI (Table 1). As shown in Table 2, a significant association was revealed between shift (*χ*^2^ = 17.64, *p* < 0.001), pressure from work (*χ*^2^ = 5.78, *p* < 0.001) and exposure to PI. In addition, the use of prescribed protocols (*χ*^2^ = 19.52, *p* < 0.001), and the use of PPE (*χ*^2^ = 8.14, *p* = 0.043) were associated with exposure to PI. Furthermore, adherence to standard precautions (*χ*^2^ = 13.73, *p* = 0.001) was related to PI exposure.

### 3.5 Factors associated with exposure to percutaneous injuries among health workers

Table 3 summarizes the bivariate and multiple log-binomial regression analyses between the predisposing factors and exposure to PI. In multivariate log-binomial regression analysis, years of experience, shift, pressure at work, and adherence to standard precautions were associated with exposure to PI. For every unit increase in years of work experience (APR = 0.97, 95% CI = 0.94–0.99, *p* = 0.021), the prevalence of PI decreased by 3%. Health workers in a mix of day, evening, and night shifts showed a higher prevalence of PI (APR = 1.69, 95% CI = 1.26–2.27, *p* = 0.001) than those in only day shifts. In addition, the prevalence of PI was higher among health workers who frequently experienced pressure at work (APR = 1.32, 95% CI = 1.00–1.75, *p* = 0.047) than among those who occasionally experience pressure at work. Lastly, a lower prevalence of PI was observed among health workers who adhered to standard precautions most of the time (APR = 0.59, 95% CI = 0.40–0.88, *p* = 0.010) and always (APR = 0.55, 95% CI = 0.40–0.77, *p* < 0.001) than those who only occasionally adhered.

**Table 3:** Factors associated with percutaneous injuries among health workers.

Characteristics	Percutaneous injuries (*n* = 602)				
	*N*	CPR (95% CI)	*p*-value	APR (95% CI)	*p*
Years of experience					
Median	5.0	0.95 (0.93–0.98)	0.001*	0.97 (0.94–0.99)	0.021*
Working days in a week					
5 and below	488	1		1	
Above 5	114	1.27 (0.93–1.72)	0.128	0.95 (0.69–1.30)	0.740
Overtime					
No	294	1		1	
Yes	308	1.22 (0.94–1.60)	0.138	1.13 (0.86–1.47)	0.386
Shift					
Day only	293	1		1	
A mix of day, evening and nights	309	1.79 (1.35–2.38)	< 0.001*	1.69 (1.26–2.27)	0.001*
Pressure					
Occasionally	349	1		1	
Frequently	253	1.38 (1.06–1.79)	0.016*	1.32 (1.00–1.75)	0.047*
Adherence to standard precautions					
Sometimes	65	1		1	
Most of the time	141	0.54 (0.36–0.79)	< 0.002*	0.59 (0.40–0.88)	0.010*
Always	396	0.53 (0.39–0.73)	< 0.001*	0.55 (0.40–0.77)	< 0.001*

*Significant variable (*p* < 0.05); CPR, crude prevalence ratio; APR, adjusted prevalence ratio.

## 4 Discussion

The study investigated the occurrence and factors contributing to PI among health workers. More than a quarter (26.9%) of healthcare workers have experienced PI at least once in the past year. Experienced workers and health professionals who either frequently or always adhered to standard precautions had a lower prevalence of PI. In addition, healthcare workers on a mix of day, evening, and night shifts and those who frequently experienced work pressure showed a higher prevalence of PI.

Our study revealed that a significant number of healthcare workers experienced PI in the past 12 months. This result was lower than that of most studies conducted worldwide. In a study conducted in Hawassa, Ethiopia, the prevalence of at least one episode of PI among HCWs was approximately 46.0% (19). A study conducted in the United States found that 57% of healthcare workers in a referral hospital had sustained PI (22). Additionally, a meta-analysis by Auta et al. (1) estimated a global 1-year prevalence of PI among healthcare workers of 36.4%. The variations in findings may be attributed to the study population and safety culture of the health facility. Nonetheless, lack of training, inadequate use of personal protective equipment, and poor working conditions may explain the high prevalence of PI among health workers (1).

According to this study, experienced health workers had a lower prevalence of PI. Our result is supported by a study conducted in Brazil, which found a decreasing trend in the rate of PI among health workers with ≥ 61 months of professional experience (23). Another study conducted in Ethiopia also showed that healthcare workers with more than 10 years of work experience were less likely to experience sharp injuries compared to those with less experience (22). These findings suggest that as healthcare workers gain more experience, they may develop better skills and practices to prevent PI (24). Moreover, it is important to provide ongoing training and education to all healthcare workers, regardless of their level of experience, to ensure the continued reduction of PI in the healthcare setting.

Further, a lower prevalence of PI was found among health workers who either frequently or always adhered to standard precautions. A study showed a decreasing trend in the rate of PI among healthcare workers who followed standard precautions, confirming the outcome of this study (25). A similar study analyzing patient safety climate and its impact on infection prevention practices found that adherence to standard precautions predicted lower rates of percutaneous and sharps injuries among healthcare workers (26). Healthcare workers who adhere to standard precautions are more likely to report sharps injuries, leading to a better understanding of reporting behaviors and improved workplace safety (22). Also, the decreasing trend in the rate of PI among healthcare workers who adhere to standard precautions can be attributed to multiple factors such as improved training, education, and awareness (24).

Furthermore, in our study, it was found that health workers on a mix of day, evening and night shifts had a higher prevalence of PI. There is limited specific data available on the prevalence of PI among health workers on different shifts. However, few studies have been conducted on the relationship between shift work and injury rates. For example, a study found that rotating shift work, including night shifts, was significantly associated with work injury (27). A similar study found that long hours and overtime, rather than specific shift patterns, were associated with increased injury risk (28). This suggests that the mix of day, evening, and night shifts may not be a direct cause of higher PI rates, but the long hours spent at work may be a reason for this higher prevalence of injury among healthcare professionals. Nonetheless, shift work often involves higher patient volumes and more complex cases during certain times, leading to increased stress and the potential for rushed or careless actions, which can result in PI.

Again, according to our findings, a higher prevalence of PI was found among health workers who frequently experience work pressure. There is limited literature specifically addressing the prevalence of PI among health workers who frequently experience work pressure. Nevertheless, a study conducted in a newly built tertiary hospital in Athens, Greece, reported a PI incidence of 3.38 per 100 full-time employment-years (FTEYs) among high-risk personnel (nursing, medical, and cleaning staff) (29). When healthcare workers are under pressure to complete tasks quickly or meet productivity targets, they may feel compelled to work hastily, bypassing safety protocols or taking shortcuts. Also, in a rushed work environment, the risk of accidental needle sticks or other PI may be heightened.

### 4.1 Implications for practice and policy

The findings of this study underscore significant implications for both practice and policy within healthcare settings. With over a quarter of healthcare workers experiencing PI annually, there is a pressing need for robust policy interventions aimed at prevention. Besides, adherence to standard precautions appears to correlate with lower prevalence rates, suggesting that reinforcing these measures could effectively mitigate risks. Moreover, the observed association between the increased prevalence of injuries and certain work-related factors such as shift rotation and high levels of work pressure highlights the need for targeted interventions. Policymakers and healthcare administrators should prioritize the development and implementation of comprehensive safety policies that not only emphasize adherence to standard precautions but also address systemic issues such as staffing schedules and workload management. By doing so, healthcare organizations can foster safer work environments and better protect the wellbeing of their workforce.

### 4.2 Strength and limitations

The research was carried out among healthcare workers chosen from 10 private and public hospitals located in the National Capital of Ghana, aiming to represent the general situation across the country. However, this study has certain inherent limitations. The utilization of a cross-sectional study design prohibits the definitive establishment of cause-and-effect relationships or the determination of the sequential order of causation among different factors. Furthermore, the study is susceptible to recall bias, as participants were required to recall events from the previous 12 months.

## 5 Conclusion

The prevalence of PI among healthcare workers in the Greater Accra region was substantial but better than some parts of the world. Experienced personnel and healthcare professionals who consistently adhered to standard precautions had a lower occurrence of PI. Conversely, those who worked across different shifts and frequently encountered high levels of work pressure showed a higher incidence of PI. Health administrators, managers and policymakers should develop and enforce workplace safety annual plans and policies that prioritize the reduction of work-related pressure and promote a culture of safety across all shifts. Future inquiries could utilize prospective cohort studies to provide empirical evidence for establishing causal relationships.

## Data Availability

The raw data supporting the conclusions of this article will be made available by the authors, without undue reservation.
